# An evaluation of primary care led dementia diagnostic services in Bristol

**DOI:** 10.1186/s12913-014-0592-3

**Published:** 2014-11-29

**Authors:** Emily Dodd, Richard Cheston, Tina Fear, Ellie Brown, Chris Fox, Clare Morley, Rosalyn Jefferies, Richard Gray

**Affiliations:** UWE Bristol, Glenside Campus, Blackberry Hill, Bristol, BS16 1DD UK; The University of East Anglia and Norfolk and Suffolk NHS Foundation Trust, Norwich, UK; Bristol Memory Service, Callington Road Hospital, Devon Partnership NHS Trust, Bristol, BS4 5PJ UK; Bristol Community Health, South Plaza, Marlborough Street, Bristol, BS1 3NX UK; Hamad Medical Corporation, Doha, Qatar

**Keywords:** Dementia, Alzheimer’s disease, Memory Clinics, Primary Care, Diagnosis, Participatory Research

## Abstract

**Background:**

Typically people who go to see their GP with a memory problem will be initially assessed and those patients who seem to be at risk will be referred onto a memory clinic. The demographic forces mean that memory services will need to expand to meet demand. An alternative may be to expand the role of primary care in dementia diagnosis and care. The aim of this study was to contrast patient, family member and professional experience of primary and secondary (usual) care led memory services.

**Methods:**

A qualitative, participatory study. A topic guide was developed by the peer and professional panels. Data were collected through peer led interviews of people with dementia, their family members and health professionals.

**Results:**

Eleven (21%) of the 53 GP practices in Bristol offered primary care led dementia services. Three professional panels were held and were attended by 9 professionals; nine carers but no patients were involved in the three peer panels. These panels identified four main themes: GPs rarely make independent dementia diagnosis; GPs and memory nurses work together; patients and carers generally experience a high quality diagnostic service; an absence of post diagnostic support. Evidence relating to these themes was collected through a total of 46 participants took part; 23 (50%) in primary care and 23 (50%) in the memory service.

**Conclusions:**

Patients and carers were generally satisfied with either primary or secondary care led approaches to dementia diagnosis. Their major concern, shared with many health care professionals, was a lack of post diagnostic support.

## Background

Dementia describes a constellation of different illnesses, the most common of which are Alzheimer’s disease and Vascular and Lewy-Body dementia. Typically most people initially experience a progressive decline in their memory, reasoning and communication skills [1]. As the illness progresses, almost all of a person’s cognitive abilities are affected, with a consequent loss of ability to perform most activities of daily living and increased dependency [2]. The illness has a profound impact on people affected by dementia and on family members who often provide the majority of care [3].

The major risk factor for all forms of dementia is age; one in 14 people over the age of 65 and one in six over the age of 80 will develop a form of the illness [1]. Dementia is recognised as a major health concern. Estimates vary but a recent study suggests the numbers of people estimated to have dementia in the UK appear to have stabilised at around 700,000 [4]. While, acetylcholinesterase inhibitors such as Donepezil (or Aricept) can be prescribed for people with Lewy-Body disease if they have distressing symptoms such as challenging behaviour, in practice, the main form of dementia for which pharmacological treatment is prescribed is mild-to-moderate levels of Alzheimer’s disease [5]. While medication can alleviate some of the symptoms, it does not alter the underlying disease process [6].

Typically people who go to see their GP with a memory problem will be initially assessed by the GP using a brief cognitive screening test of their memory. This is not diagnostic, but suggests impaired memory. Patients are referred to a memory clinic for a more a comprehensive assessment and ultimately a diagnosis. Memory clinics are multidisciplinary teams that typically include psychiatrists, memory nurses, and clinical psychologists. These were introduced in the 1980s, often with a specific research focus [7,8]. The first anti-dementia drugs in 2001 spurred the evolution of a national network of memory clinics, at least in part because NICE (The National Institute for Health and Care Excellence) recommended these drugs should only be prescribed following a specialist assessment [8]. An influential study [9] showed that memory clinics were effective at improving recognition of dementia and enhancing the quality of life of both patients and their carers. However clinics have been criticised for not paying enough attention to post-diagnostic support even though different forms of post-diagnostic care have been described [10,11].

Demographic forces mean that there will be pressure on memory services to continue to expand to meet demand as the population ages. This is problematic for two reasons: first, there are a finite number of clinicians (particularly old age psychiatrists, clinical psychologists and memory nurses) with the skills required to work in these services; second, expanding memory clinics will require additional funding, which is challenging at a time of austerity. Finally, policy drivers within the health service on timely dementia diagnosis only serves to add to this burden [12].

### Primary care and dementia

An alternative model might to develop the role of primary care making routine dementia diagnosis. The involvement of GPs in the process of assessment, diagnosis and treatment of dementia, would have a number of potential advantages: not only is primary care ideally placed to recognise and manage dementia as the first point of call and the gateway to other health and social support services, but because of their often close and well-established relationship with patients, GPs may be in the ideal position to recognise the early signs of a possible dementia, and to initiate appropriate assessment and treatment plans. Underpinning the potential involvement of primary care in making a dementia diagnosis is evidence that GPs are reasonably accurate at detecting, in particular, moderate to severe dementia and respond well to training in detection and management of the illness [13,14].

At the same time, the barriers to effective service provision for people with dementia in primary care have been well documented [15]. In addition to patient and community barriers that restrict the initial identification of problems by the medical system, there are also other barriers to effective diagnosis. Most GPs tend to have relatively little experience of working with people with dementia. For instance in the UK, a typical GP might expect to have only one or two patients every year diagnosed as having dementia, and have between twelve and fifteen patients at any one time with this condition [16]. Similarly, primary care physicians often cite the absence of ring-fenced time as a major factor leading to their inability to provide a specific dementia service [17,18]. Lack of knowledge amongst GPs about dementia, and a reluctance to make a diagnosis can be particularly problematic for people from specific groups, such as people with learning disabilities or younger people with dementia [19].

### Primary care led dementia services

Although at least two other service frameworks have been described in which assessment takes place in primary care in the UK, in both instances this has been due to specialist staff coming into primary care, rather than the primary care health team themselves developing new skills. The model adopted in Gnosall involved integration of a monthly memory clinic within every GP practice [20]. A consultant psychiatrist was available for contact at all times and main coordinator of care was appointed, labelled as the ‘eldercare facilitator’ [21]. A second model has been described in Croydon [9]. Unlike the Gnosall model, the Croydon model was a complementary service involving professionals from health, social and voluntary services within the area to provide individually-tailored approach of assessing and managing people with dementia. All professionals within the services receive the same training about dementia and its management, and their role is to assist early recognition and assessment of people with suspected dementia.

#### Local service development

In Bristol, a city with a population of just under half a million, of which 60,000 are aged over 65, a model of primary care led dementia services was piloted in eleven primary care practices between August 2012 and December 2013. Each practice that volunteered to take part in the pilot received a payment of £1, 000 for involvement, and additional bonus payments were given for a 5% increase in the number of dementia diagnoses. The percentage of people aged over 50 registered with the pilot practices (31.6%) is comparable to the non-participating practices (28.5%). Three memory nurses from the secondary care memory service were seconded to work with the eleven pilot practices. As part of the pilot programme, participating GPs attended a three-hour training session delivered in their practice by the GP lead for dementia and the memory clinic service manager which focussed on identifying, assessing and diagnosing dementia.

During the pilot study, the remaining 44 Bristol practices continued the previous service model, in which all patients suspected of dementia symptoms were referred to the secondary care memory clinic for assessment and diagnosis. This involves a memory nurse of psychology assistant meeting the patient and family to collect a clinical history and undertake cognitive assessment. The formulation alongside results from CT scan and blood screen are discussed with a specialist doctor and a diagnosis reached. However, following an evaluation of the pilot project, in January 2014, the model was rolled out so that all GP practices in the Bristol area were working under the new guidelines. Central to this approach is that primary care practitioners no longer automatically referred all patients with suspected memory problems to secondary care for assessment. Instead a stepped approach was adopted in making a dementia diagnosis, so that wherever possible the diagnosis was made in primary care. The thinking that underpins this approach was that in some cases it is clear that patients are experiencing significant memory difficulties, and the range of alternative explanations for their memory loss and other cognitive changes can be excluded without the need for specialist referral.

Where diagnosis is more equivocal, GPs can seek advice from experienced memory nurses located in primary care, either in person or over the telephone. Patients where a diagnosis is complex or uncertain can then still be referred to the memory service for a comprehensive diagnostic assessment. The overall aim of the Bristol primary care led model of dementia services is to ensure that patients receive an accurate and timely diagnosis within primary care. For those patients where referral to secondary care is indicated, then this could still be expedited as the demand on secondary care services had been reduced.

## Methods

The objective of this service evaluation was to contrast participants’ experiences of primary care led dementia services in Bristol with existing secondary care based memory services. We adopted a participatory approach and sought to interview people with dementia their carers and the health professionals that provided their care and treatment. We strictly adhered to COREQ (Consolidated Criteria for Reporting Qualitative research) standards of reporting [22] and RATS (Relevance, Appropriateness, Transparency and Soundness) guidelines [23].

### Theoretical framework

We sought to collaborate with people with dementia, their family and friends and those working in health and social care services. This participatory approach enabled an in-depth understanding of both models of dementia diagnosis and care from a range of different perspectives (e.g. patient, family, professional).

### Research team and reflexivity

The research team were a mixture of males and females, of a range of ages and were educated to at least degree level or above. Most had recent secondary care clinical experience of working with people with dementia. The research team included psychiatric nurses (CM, RJ, RG), a primary care nurse (TF) psychologists (RC, EB), a project manager (ED) and an old age psychiatrist (CF).

Seven peer interviewers were recruited to carry out the interviews - all of whom had personal experience of caring for a family member with dementia. All of the peer researchers were female with an average age of 62 years. Criminal Records Bureau (CRB) checks were completed for all peer interviewers.

A one-day training programme was provided to ensure that peer researchers were confident and competent in conducting interviews with people with dementia, family members and health professionals. All interviewers were paid for the hours they worked and were reimbursed travel expenses. Peer interviewers may confer a number of advantages over professional researchers; they have a unique perspective on caring for someone with dementia, and their caring experience may enable them to be more empathic when conducting interviews.

### Professional and peer panels

We held three professional and three peer panel meetings over the course of the study. There were seven (six female, one male) members of the professional panel, a GP, a psychiatrist, a nurse specialist, 2 memory nurses, a care home manager, and a third sector manager (who service provides post diagnostic support). The peer panel comprised nine members all of whom provided care to a family member with dementia. Despite a number of attempts through memory cafes, we were not successful in recruiting someone living with dementia to the panel. The professional and peer panels enabled us to develop a framework or topic guide which was the basis of the interviews which were carried out with participants.

### Sampling

Cases were people of any age who had been recently diagnosed (within the previous six months) with any form of dementia and were able to give consent to take part. People with a diagnosis of Mild Cognitive Impairment were excluded from the study. We aimed to recruit a total of sixty cases; thirty from the eleven GP practices who were piloting the primary care led dementia care pathway (ten patients with dementia, ten relatives and ten staff) and thirty from secondary mental health services (ten patients with dementia, ten relatives and ten staff).

#### Primary care

Patients were identified by directly contacting GPs in the pilot practices offering primary care led dementia services. Each pilot practice was contacted by RG to introduce the evaluation and explain what we required them to do. We then emailed the lead GP in the practice a template of an invitation letter and reply slip. We also mailed stamped addressed return envelopes (SAE). We asked GPs to send out invitations (with a SAE) to all patients in their practice who had received a dementia diagnosis in the last six months. Reply slips were returned to the University and a member of the project team (ED/RJ) telephoned the patient and/or their relative to explain the study and to arrange for a peer interviewer to visit. Following this conversation written information about the study was also sent to participants.

GPs were invited by email to participate in the study. If they indicated they were happy to take part, ED sent them an information sheet about the study and arranged a time for the peer interviewer to visit.

#### Secondary care

We identified patients in secondary care memory services by asking nurses, psychologists and psychiatrists to identify patients who had received a dementia diagnosis in the past six months. These secondary care staff then telephoned patients and/or relatives to find out if they were interested in participating in a study about their experiences of receiving a dementia diagnosis. Once verbal consent had been given, a member the project team (ED/RJ) telephoned potential participants to explain the study and book an appointment for a peer researcher to visit. Following this conversation written information about the study was also sent to participants.

To recruit health care professionals in secondary care the project coordinator (ED) attended team meetings to explain the purpose of the study and to invite memory service staff to take part. Those who expressed an interest were sent an information sheet and an interview was booked.

### Ethics approval

Ethical approval was obtained from the ethics committee of the University of the West England (Reference number: HLS-12-10-114). The research and development departments at Avon and Wiltshire Partnership NHS Trust and the Avon Primary Care Research Collaborative also approved the study as a service evaluation.

### Interview guide

Professional panel members generated questions that were incorporated in an initial draft of the interview guide. This draft was reviewed and extensively modified at subsequent meetings. The peer panel then reviewed this second draft making some further amendments. Questions were organised under four main themes; GPs making an independent dementia diagnosis; GPs working with memory nurses; patients and carers experience; post diagnostic support. We did not pilot the interview guide.

### Setting

All interviews were conducted in a location chosen by the participant. Generally, for patients and their relatives, this was their own home, while for professionals it was their place of work (e.g. GP practice, memory clinic). The University lone working policy was adhered to when interviewers visited participants.

### Presence of non-participants

On three occasions, patients and their relatives were interviewed at the same time. The peer interviewers reported that they did not feel that it was appropriate or possible to conduct the interviews separately. All other interviews were conducted on a one-to-one basis.

### Duration of interviews and number of repeat interviews

On average interviews lasted 20 minutes and varied in duration from 10 to 44 minutes. No repeat interviews were carried out.

### Audio recordings

Interviews were audio-recorded using digital recorders. In order to comply with the Data Protection Act 1998, recorders were returned to the project coordinator after each interview and the interview deleted from the device. Recordings were stored on a secure, encrypted SharePoint site, password protected and accessed by the transcribers and evaluation team only. This was to ensure safe data transfer of the recordings and transcriptions between the transcribers and evaluation team. Transcribers were existing University employees who had signed confidentiality agreements. All identifiers were removed from the transcripts to maintain anonymity and confidentiality. Transcripts were not sent to participants for checking.

### Field notes

Peer interviewers did not keep field notes but were encouraged to keep reflective diaries. The evaluation team stressed during training that if they had any concerns following an interview then they should contact the evaluation team to talk these through. In addition, the group came together on a further two occasions for group supervision and encouraged to use the support of each other.

### Description of sample

Table 1 shows the number of people with dementia, their relatives and health care professionals who participated in the evaluation. In total 46 interviews were completed. We recruited and interviewed an equal number of participants in both arms of the evaluation. There was an even number of female (n = 7, 54%) and male patients who, bar one, were all over the age of 75. There were slightly more female carer/relatives (n = 9, 60%) in the sample. The majority (n = 12, 67%) of health care professionals were female. Everyone that was interviewed across all three groups defined themselves as being of white European ethnic origin. Due to the emphasis of this study on the patients’ and relatives’ experience of the services offered, we did not feel it necessary to probe the patient or relative on the exact diagnosis given or consult medical records for this information.

**Table 1:** **Number of participants/non-participants in the study**

	**Primary care**		**Secondary care**	
	**Approached n (%)**	**Participated n (%)**	**Approached n (%)**	**Participated n (%)**
Health care professionals	19 (28)	10 (43)	9 (29)	8 (35)
Patients	25 (37)	6 (26)	11 (35)	7 (30)
Carer/relative	24 (35)	7 (30)	11 (35)	8 (35)
Total	68	23	31	23

## Results

Using a prescribed process [24], four members of the research team (TF, EB, ED, RG) undertook the data coding, identifying evidence that related to each of the four main themes from the panel meetings. Following each quote we have indicated if the respondent is: in the (PC) primary care or (SC) secondary care arm of the study and if they are a (P) patient, (C) carer, or (HCP) Health Care Professional.

### Theme 1: GPs rarely making independent dementia diagnosis

GPs that participated in this evaluation were, on the whole, cautious about making a dementia diagnosis independently. In part this seemed to be because GPs don’t often “*see new patients with dementia coming through*” [PC, HCP]. Each pilot practice was asked how often they consulted with or referred patients with memory problems to a memory nurse, seven (66%) responded. Three reported that they would send almost all of the patients they saw with memory problems to the memory nurse (who they viewed as an additional resource to the practice) for assessment. When asked almost all relatives and patients commented on having a visit from a memory nurse except one patient:

*Patient: “No I didn’t go to see anybody else I only seen the doctor.”*

*Interviewer: “No have you been to see anybody has your doctor sent you to a clinic say to see somebody?”*

*Patient: “No.”*

*Interviewer: “You haven’t seen anybody?”*

*Patient: “No...”* [PC, P]

Use of the memory nurse was more considered by two GPs who said they would make some independent diagnoses but would also consult with or make a referral to a memory nurses when they felt this was required. Two GPs said that generally they would make a dementia diagnosis independently, only referring patients with specific presentations to the memory service. These would include younger patients, those who they thought might have Lewy body syndrome and those with behavioural problems.

### Sub theme: a lack of confidence

A lack of confidence seemed to be a major barrier to GPs taking a more active lead in making a dementia diagnosis. Talking about being confident in making a diagnosis independently, one HCP said only when it was a “*barn door dementia*” [PC, HCP], another that it was “*not an easy diagnosis to make*” [PC, HCP]. There was a certain sense that as the pilot project has progressed, GP confidence has developed. For example one GP said, “*I feel a lot more competent than I did a year ago when the pilot started*” [PC, HCP]. Two said that they were “*Feeling more confident and were less reliant on memory nurses*” [PC, HCP and PC, HCP] whilst another felt that confidence will grow “*I think we do need the support, we may need less support as time goes on”* [PC, HCP]. One GP reported considerable confidence in making a dementia diagnosis and would do this for about “three quarters of the patient’s I see with memory problems” [PC, HCP].

Training was often referred to by GPs as one of the factors that enhanced their confidence in making diagnoses. The chance to network and discuss the pilot was particularly valued by the HCPs interviewed.

*“…reinforced the protocol and the prescribing and everything and everyone found that very useful…. The best talks were from GPs with special interests or people who knew what it was like in general practice”* [PC, HCP].

*“I think the workshop and the training that I went to were very good, very targeted and with the opportunity for questions. I do think perhaps this sort of once annual…sort of annual training session”* [PC, HCP].

### Sub theme: not rushing to diagnose (screening)

One of the major concerns expressed at the professional panel meetings was that GPs would make a dementia diagnosis based solely on information from screening tests (e.g. GP cog), running the risk of an inappropriate diagnosis being made. From the interviews that were completed this did not seem to be the case. GPs reported that in addition to using screening tests they also carried out a relatively thorough medical history and certainly considered alternative causes for memory problems. GPs had a modal average of around four consultations with patients (and carers) before they made a dementia diagnosis.

The GPs interviewed all commented that they do not actively screen but would respond to patients who have concerns about their memory:

“*At the moment we don’t have any proactive screening. We would … we would respond in the surgery anyway to people who come and report memory problems…if they came and told us they had a memory problem then we would respond to that… But I don’t call that screening”* [PC, HCP].

### Theme 2: GPs and memory nurses working together

From a GP perspective, when he or she sees a patient that they suspect has dementia they will discuss this with the memory nurse attached to their practice, either over the phone or at lunch time meetings with other GP’s in the practice. This was in line with the pilot project dementia care pathway that states there should be a GP discussion with a memory nurse before a diagnosis is made. Memory nurses found liaising with GPs to be cumbersome and time consuming. One nurse for example said “[it] *takes up a lot of time. Getting hold of GP’s, responding to emails*” [PC, HCP].

Despite this, GPs reported that they value this working relationship with the memory nurse:

*“I like discussing patients with the memory nurse”* [PC, HCP].

Although some GPs use the memory nurse for advice only, most directly referred patients to them for assessment. This was clearly evident from the interviews with memory nurses, patients and relatives:

*“Um and we had a visit you know from um… the GP arranged a visit from a memory nurse and she came and saw us and she was absolutely excellent wasn’t she?”* [PC, P].

*“With pilot people it is slightly different because I don’t have the memory clinic doctor…I’d be seeing people in their own home…same cognitive testing like we do here but really kind of cutting it down and simplifying it”* [PC, HCP].

One nurse felt that this role she had taken on within primary care seemed to be more time consuming than when working in the memory clinic:

*“I feel a lot busier…but I don’t know if I am seeing quite as many people as I would have done”* [PC, HCP].

### Theme 3: patients and carers generally experience a high quality diagnostic service

Responses from patients and carers suggested they did not notice a difference between primary and secondary care diagnostic pathways and generally, most gave positive accounts of their experience and of the health professionals that they encountered:

“*It was all dealt with really quickly…I don’t think that they could have done much better than they did and it was swift and informative*” [SC, C].

*Interviewer: “Now I know it’s quite hard isn’t it to remember the passage of time, but can you think if you had to wait a long time for…”*

*Patient: “Oh no, that was a… I’m surprised how quick it was [quite quick].”*

*Interviewer: “that’s good then, so you’re quite impressed with that then?”*

*Patient: “Oh yes, I got no complaints there. [no].”* [PC, P]

However there were a couple of carers who felt the process was not streamlined enough:

*“it was delay, delay, delay”* [SC, C].

*“Eventually yes. There were long delays”* [SC, C]*.*

It was striking how valued memory nurses were:

“*I can’t fault {…}, who came round immediately had my mum at ease, taking about the children, grandchildren*” [PC, C].

Those participants that were referred to the memory service spoke little about any diagnostic input from their GP, but their importance at the beginning of the diagnostic process was acknowledged:

“*I guess it all starts with the GP doesn’t it, it all depends on your GP because that’s who starts the ball rolling*” [SC, C].

This perhaps emphasises the important gatekeeping role of the GP. Many of the carers spoke of GPs not always taking action when concerns were first raised but instead told them to “*keep an eye on it and in a couple months if it is still the same we’ll refer her for tests*” [SC, C].

One carer taking the primary care pathway, particularly felt she wasn’t being listened to by the GP:

*“… she’d gone out at night time wandering, umm when that was brought to my attention I thought really I’d best get the doctor involved. So called her GP and explained to her and she came to the house. And after she’d been to the house I asked if any meetings, any dealings I could be informed, gave them my number and I wasn’t informed then but a nurse, a district nurse came round, questioned my mum and when I took my mum back to see the doctor, the doctor informed me there was nothing wrong with mum*” [PC, C].

There was a sense from a couple of the carers particularly that they felt they had been “fobbed off” and that memory problems were not taken seriously by GPs.

*“I thought, this is ridiculous, I’m taking mum for memory problems, why is the GP phoning my mother? Why not me? … So I felt it was just … that they weren’t really understanding what the problems were”* [SC, C].

### Sub-theme: diagnostic practice in the memory service

Clinicians who worked in secondary care seemed to be, by and large, adherent with the secondary care dementia diagnostic pathway. One memory nurse described how this involved:

“*going through a detailed clinical history, how did the memory problems start? … go through lots and lots of different areas of cognition and thinking … all those things and then I would do some cognitive testing with them*” [SC, HCP].

A key part of the secondary care pathway is the memory nurse being able to discuss the results with a consultant psychiatrist and being able to feedback the results to the patient the same day. The family spend a number of hours with the clinic team on one day and at the end of this process a diagnosis is provided. This is in contrast to the primary care process of reaching a diagnosis over the course of maybe four, ten-minute consultations:

*“…it’s quite tricky because we only have ten minutes so it’s really hard and you can’t keep bringing people back so I suspect we do make it [the diagnosis] on very quick honed criteria and gut instinct which is hard to define”* [PC, HCP].

This model of consultative team working was not present in the primary care arm of the study. For those in secondary care, patients whose diagnosis is uncertain or where there is multi-morbidity are referred by the psychology team. The assessment provided at this point will test “*problem solving, planning and sequencing skills, visual spatial skills and concentration and attention*” [SC, HCP].

The outcome of this assessment may vary, and can help to exclude a diagnosis of dementia. For example “*It’s often a case of people having low mood or depression and in which case I might recommend counselling or having anti-depressant medication*” [SC, HCP].

The expertise of secondary care clinicians was emphasised by health care professional working in both secondary and primary care. Concern about possible mis-diagnosis in primary care was expressed:

“W*ithin core this is a specialist service and we try very hard to get the diagnosis exactly right and [primary care led dementia services are] trying to simplify that … with the main emphasis on making sure you can pick out the people who are complicated…”* [SC, HCP].

“*…what about those ones who seem straightforward, are they really straightforward or actually are there things that were missing which if they had gone through this proper process might have been picked up…*” [PC, HCP].

### Sub-theme: communication about diagnosis

Being given a dementia diagnosis will have a major impact on the lives of patients and their relative. This was reflected in what carers reported in their interviews, for example one carer said “*so I had my fingers crossed but they said it was Alzheimer’s and I was gutted*” [SC-C].

Giving patients a clear diagnosis in a sensitive and compassionate manner was described by memory nurses in the professional panel meetings as being very important. They talked about devoting considerable time and effort to ensuring patients/carers understood the illness and treatment. This contrasts with primary care practice where carers’ accounts seem to indicate that less emphasis was placed on giving a diagnosis. Indeed there was some suggestion that GPs may have avoided telling patients and their carers that they had dementia:

“…*he (GP) just gave me (mother) tablets in a blister pack but I don’t know what they are”* [PC, C].

For many of the carers getting a diagnosis was considered to be a relief;

“*I felt relieved actually*” [PC, C].

*“Actually it was a huge relief when we got the diagnosis to be honest…because we know what we were dealing with”* [SC, C].

*“The thing is if you know you’ve got a problem, you know it’s an ongoing thing and rather than have a big upheaval you can change things gradually*” [SC, C].

*“it’s important to know if it is an illness…you can get help and I am hoping that these tablets will just prolong before it gets steadily worse so yeah it is important to get it sorted out*” [SC, C].

One patient who had been diagnosed with early onset dementia reflected on the process of being given the diagnosis:

*“So yeah as I say it’s been pretty reasonable but in terms of the giving information I think it would be good particularly if you are early onset to yes to give them diagnosis and some initial information but then set up a session to then have another maybe more general conversation a few weeks later because they can take the step back a little bit more”* [SC, P].

In addition, although most of the carers felt that there was no real choices offered but medication, in many instances, pills were seen as positive for both the patient and the carer:

“*last year …we were away every weekend on our motorbike…I didn’t think I was going to be able to do anything now because I need to check on mum all the time but in the last couple of weeks it has really changed. [Since] she’s been on the medication there are signs she is calmer*” [PC, C].

“*he’s a little bit more with it…found a new belt [for the vacuum cleaner], put the thing on and…I’ve been using it ever since. Well yeah he couldn’t have done that before*” [SC, C].

What was clear was that the process of supporting patients through the assessment was much more thorough in secondary compared to primary care. At the same time, primary care patients did not report that they had experienced being pushed through the assessment in a rushed or inappropriate way.

### Theme 4: an absence of post diagnostic support

Carers reported varying degrees of follow up support that was offered. The focus of support tended to be for the patient rather than the carer. Any information came either from memory nurses and other memory service staff or through the media such as TV or newspapers. No carers or patients said explicitly that they had received any support from the GP, although a couple felt they could ring and speak to the GP if needed. Most of the support on offer was signposting to services for practical help; adaptations to the house via social services, council tax rebates, arranging power of attorney. When voluntary sector support such as Alzheimer’s Society memory cafes or Singing for the Brain was suggested by the interviewers, one patient responded positively about their experience:

*Patient: “Yeah we went to the Memory Café recently.”*

*Interviewer: “Ok and what did you think of that?”*

*Patient: “Nice.”*

*Interviewer: “What was good about it?”*

*Patient: “Well it was just nice and the company as well they were all very bright and jolly and its just nice to sit and relax yeah and chat yes”* [SC, P].

But most participants had not heard of these avenues of support. Many relatives cited several reasons why they thought their loved one would not take this up:

“*but she did say ‘oh there’s a coffee place at there’ and I said to {…}, he said no…I think its all you know, it was labelling him*” [SC, C].

“*I phoned the memory clinic because they’re so helpful, they gave me the number of social services and said she may be entitled to some help. But just at the moment mum doesn’t want anyone else to come in*” [PC, C].

There was a sense from the interviews that the focus in both primary and secondary care models is on diagnosis with little in the way of robust post diagnostic support, either for the patient or carer:

*“I had rather hoped that we might get some advice but you know in January I must say it didn’t seem likely”* [SC, C].

*“Well I don’t think we were given any support really no…I would have liked to have been told about the various groups that are there to help”* [SC, C].

*“It’s a nuisance and I was hoping that there’d be something that they would say well do this and you know…oh I started doing crossword puzzles [okay] and what’s those other things, Sudoko’s I can’t understand those”* [PC, P].

This was also highlighted as a concern by staff across primary care:

*“I probably feel…probably feel more responsible in terms of they are still my people when they come into core but I feel more worried about letting people in the pilot project go because I know nobody else is going to do that”* [PC, HCP].

“*…most of us would like to be involved in that ongoing hand holding throughout the person’s journey”* [PC, HCP].

## Discussion

The overall aim of this study was to use a participatory approach to contrast patients, carers and health care professionals’ experiences of a primary and secondary care (memory clinic) led dementia care pathway. In total 46 interviews were completed by peer researchers, complemented by evidence from professional and user panels. Perhaps our most important observation was that patients and carers generally found either primary or secondary care dementia pathways to be acceptable. This is an important observation as previous evaluations of memory services have used patient acceptability as one of the predefined service goals [9]. It was interesting to note that many of GPs in the pilot did not feel able to conduct a dementia assessment without involving a memory nurse. There was some promise that as the pilot progressed GPs were becoming more confident in making a diagnosis themselves. There was concern that diagnosing in primary care may mean people being diagnosed erroneously. Other studies suggesting that the opposite is true and primary care clinicians are more conservative in their dementia diagnosis for a number of reasons; practitioner lack of confidence and associated risk averseness, therapeutic nihilism, negative attitudes toward the potential benefits of detecting and managing dementia [16,25,26].

Regardless of the potential benefits of assessing people within a primary care setting, there are also some risks which this evaluation did not address. The level of cognitive assessment in primary care, for instance, is largely confined to screening tests rather than the more comprehensive assessment that are common in specialist memory clinics. Clinical judgement can be particularly difficult in the early stages of dementia. Poor hearing and/or vision, a lack of education or learning disability, poor physical or mental health, different culture and language can all lead to overestimates of cognitive change. Equally, a person of very high ability may pass a basic screening test and consequently, subtle cognitive difficulties that are important for diagnosis, may be missed. All such cases need a specialised cognitive assessment by a neuropsychologist or equivalent specialist [27].

There is a clear emphasis on gaining informed consent during the assessment and diagnostic process in secondary care memory clinics. Memory clinics accredited through the Memory Services National Accreditation Programme (MSNAP) adhere to a number of set standards, one of which details “clear procedures for gaining consent and ensure that people with memory problems/dementia are well-informed of their rights regarding consent” ([28] p.22). There is concern that this standard alongside other good practice measures adopted in secondary care are absent when assessing and diagnosing dementia in primary care just by the very nature of the informality of working practices in that environment.

Participants were uniform in the praise of the work of memory nurses in both primary and secondary care settings. Very little research has been published about the role nurses working in memory services play; most evaluations focus on the specific diagnostic elements of the service. It was striking from the interviews how valued they were by patients, carers and GPs. Certainly if the primary care dementia pathway is to continue then the expert consultancy they provide to GPs would seem to be important.

The paucity of post-diagnostic support was an important theme in our study. Our findings are consistent with other studies that have drawn attention to a lack of support following a dementia diagnosis [29]. Only those people who are given a diagnosis of Alzheimer’s disease are routinely offered follow up (in order to monitor medication). Post-diagnostic support groups are provided within local secondary care but not everyone diagnosed by the service will be referred onto these. This is in contrast to other memory services which do include post-diagnostic counselling and adjustment as well as psychosocial interventions such as Cognitive Stimulation Therapy [30]. Given this variation of secondary care models across the country limits the generalisability of this study’s findings to other areas Many people affected by dementia receive little or no further support from the health service, but are routinely advised to contact the third sector (e.g. the Alzheimer’s society) for support. The absence of a coherent post-diagnostic pathway that is adequately funded means that for many people the opportunities for adjustment that are afforded by an early diagnosis will be missed.

### Limitations

That only a quarter of the patients and carers in the primary care arm of the study invited to take part agreed to participate is an important limitation. It is possible that patients and carers who were more satisfied with the service they had received were more likely to agree to participate in the study. That we relied on HCPs in memory clinics to assist with recruitment in this arm of the study is also a limitation. HCPs might have been more likely to “hand pick” participants for the study, either because they felt they were more likely to consent to take part or because they believed they were more likely to say favourable things about the memory service.

Our use of peer researchers to conduct the interviews could be considered a constraint. There was some evidence that when conducting interviews they were prone to ask leading questions eg “*I suppose [carer] is feeling better because he can see an improvement in you?*” and missed prompts from participants to pursue a line of questioning. It was evident from the transcripts that peer interviewers were more comfortable to interview and engage in conversation with the relatives than with people living with dementia. This is not surprising considering they came with their own experience of caring for a loved one. Interviews with participants diagnosed with dementia were shorter and were styled towards a more structured question/answer type interview. On occasion the relative was present in patient interviews when it was evident that both the person with dementia and the interviewee at times looked to the relative to assist in answering the question. This can be seen as a limitation of the training rather than on the abilities of the interviewers themselves. Interview techniques and role plays should have also included examples of interviewing people with dementia as well as interviewing relatives. That said, we chose to work with peer researchers because we believed that patients and carers would be more likely to open up to someone who had experience of living with dementia. The detail and richness of the responses that participants gave when being interviewed lends weight to this approach.

Efforts were made to invite people with dementia to take part as peer researchers and/or panel members through local memory cafes but there was difficulty in either subsequently contacting interested parties to take part or the invitation was declined.

Given the variation of secondary care models across the country limits the generalisability of this study’s findings to other areas. It does though offer useful insights.

The focus of this study on the acceptability of a primary care dementia pathway and not on diagnostic accuracy by GPs is a limitation. The possibility that GP diagnoses were less accurate than those arrived at by memory clinics was not one that we could test given the methodological approach we had adopted. It would be informative if future studies could explore the effect of training GPs in dementia on diagnostic accuracy in different stages of dementia.

## Conclusion

Patients and carers were generally satisfied with either primary care or secondary care led approaches to dementia diagnosis. Their major concern, along with many health care professionals, was a lack of post diagnostic support. The variation of secondary care models across the country limits the generalisability of this study’s findings to other areas. It does though offer useful insights into how and what patients, carers and health professionals using and working in secondary care and primary care services think and feel about the evolving dementia diagnosis pathway in Bristol.
